# A Rosetta Stone for Nature’s Benefits to People

**DOI:** 10.1371/journal.pbio.1002040

**Published:** 2015-01-13

**Authors:** Sandra Díaz, Sebsebe Demissew, Carlos Joly, W. Mark Lonsdale, Anne Larigauderie

**Affiliations:** 1 Instituto Multidisciplinario de Biología Vegetal (IMBIV- CONICET) and FCEFyN, Universidad Nacional de Córdoba, Córdoba, Argentina; 2 National Herbarium, Department of Plant Biology and Biodiversity Management, College of Natural Sciences, Addis Ababa University, Addis Ababa, Ethiopia; 3 Departamento de Biologia Vegetal, Instituto de Biologia, Universidade Estadual de Campinas, UNICAMP, Campinas, Brazil; 4 Commonwealth Scientific and Industrial Research Organization, Canberra, Australia; 5 Intergovernmental Platform on Biodiversity and Ecosystem Services Secretariat, UN Campus, Bonn, Germany

## Abstract

After a long incubation period, the Intergovernmental Platform on Biodiversity and Ecosystem Services (IPBES) is now underway. Underpinning all its activities is the IPBES Conceptual Framework (CF), a simplified model of the interactions between nature and people. Drawing on the legacy of previous large-scale environmental assessments, the CF goes further in explicitly embracing different disciplines and knowledge systems (including indigenous and local knowledge) in the co-construction of assessments of the state of the world’s biodiversity and the benefits it provides to humans. The CF can be thought of as a kind of “Rosetta Stone” that highlights commonalities between diverse value sets and seeks to facilitate crossdisciplinary and crosscultural understanding. We argue that the CF will contribute to the increasing trend towards interdisciplinarity in understanding and managing the environment. Rather than displacing disciplinary science, however, we believe that the CF will provide new contexts of discovery and policy applications for it.

## Abstract 2

A new conceptual framework for biodiversity and ecosystems sets out to connect scientific concepts and the human experience of nature, bridging the gaps between different value systems.

## Introduction

The Intergovernmental Platform on Biodiversity and Ecosystem Services (IPBES) [1] (www.ipbes.net), was established to strengthen the science–policy interface for the conservation of biodiversity, ecosystem services, long-term human well-being, and sustainable development. It is similar to the Intergovernmental Panel on Climate Change (IPCC) in that it will carry out assessments of existing knowledge in response to governments’ and other stakeholders’ requests [1]. However, the challenges for IPBES are arguably more complex [2] because, although the biodiversity crisis is global, biodiversity distribution and its conservation status is hugely heterogeneous across the planet; therefore, the solutions will have to be scalable to a much finer level, and the relative contributions of such fine-scale solutions to the improvement of global biodiversity status will also vary enormously.

IPBES has three distinctive features. First, it must engage, in the process of defining questions, assessing trends, and identifying solutions, a great diversity of stakeholders including policy makers, practitioners, civil society organisations, and the private sector. Second, it aims to incorporate knowledge from a variety of sources, including not only the natural, social, and engineering sciences but also indigenous and local knowledge (ILK). The inclusion of ILK is not only a matter of equity but also a source of knowledge that we can no longer afford to ignore [3–5]. Third, IPBES goes beyond producing assessments to include capacity-building, development of policy tools, and catalysing the generation of critical new knowledge.

A conceptual framework was required to give cohesion to this ambitious vision. Such scaffolding needed to provide an integrated view of the biodiversity knowledge–policy interface, stimulate new thinking, accommodate diverse human attitudes to biodiversity, and at the same time be as simple as possible to be effective and useful for the diverse array of stakeholders. The conceptual framework (CF) adopted by IPBES rises to this challenge. In this piece, we briefly summarise its main features and argue for its potential to improve the science–policy interface and also advance fundamental science on the links between biodiversity, ecosystems, and societies.

## A Framework for the Knowledge–Policy Interface on Biodiversity and Its Societal Benefits

**Figure 1:**
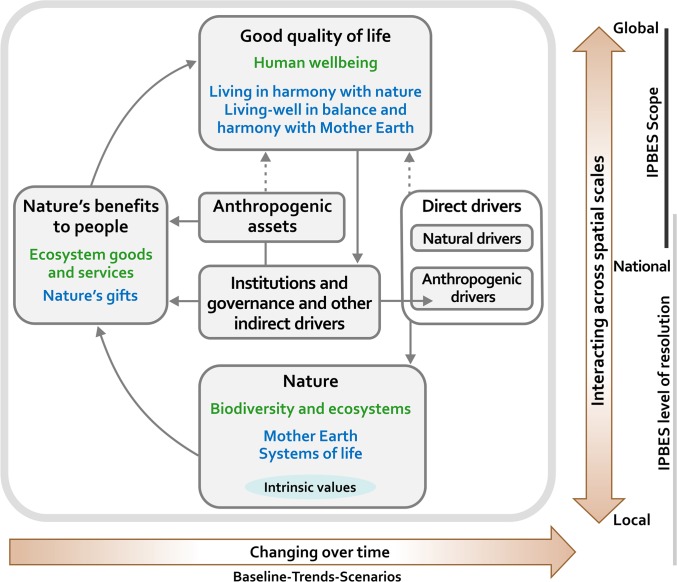
The IPBES Conceptual Framework. In the central panel, delimited in grey, boxes and arrows denote the elements of nature and society that are at the main focus of the Platform. In each of the boxes, the headlines in black are inclusive categories that should be intelligible and relevant to all stakeholders involved in IPBES and embrace the categories of western science (in green) and equivalent or similar categories according to other knowledge systems (in blue). The blue and green categories mentioned here are illustrative, not exhaustive, and are further explained in the main text. Solid arrows in the main panel denote influence between elements; the dotted arrows denote links that are acknowledged as important, but are not the main focus of the Platform. The thick, coloured arrows below and to the right of the central panel indicate that the interactions between the elements change over time (horizontal bottom arrow) and occur at various scales in space (vertical arrow). Interactions across scales [8], including cross-scale mismatches [19], occur often. The vertical lines to the right of the spatial scale arrow indicate that, although IPBES assessments will be at the supranational—subregional to global—geographical scales (scope), they will in part build on properties and relationships acting at finer—national and subnational—scales (resolution, in the sense of minimum discernible unit). The resolution line does not extend all the way to the global level because, due to the heterogeneous and spatially aggregated nature of biodiversity, even the broadest global assessments will be most useful if they retain finer resolution. This figure is a simplified version of that adopted by the Second Plenary of IPBES [21]; it retains all its essential elements but some of the detailed wording explaining each of the elements has been eliminated within the boxes to improve readability. A full description of all elements and linkages in the CF, together with examples, are given in [20].

The first public product of IPBES, the CF was constructed through more than two years of consultative work involving specialists from different sciences and knowledge systems, and was submitted to open comments by more than 100 governments and numerous nongovernmental organisations. It captures the relationships between the natural world and humankind in only six main elements—nature, nature’s benefits to people, anthropogenic assets, indirect drivers of change (such as institutions and governance systems), direct drivers of change, and good quality of life (Fig. 1, Box 1). This model clearly builds on the highly influential Millennium Ecosystem Assessment [6,7], which contemplated the essence of most of these elements and their links. However, the CF further emphasizes the crucial role of human institutions as sources of both environmental problems and solutions. Taking advantage of the remarkable conceptual and methodological progress made in this area since the early 2000s [8–13], it also goes further in its intent to consider a whole range of values from monetary to spiritual and from instrumental to relational, in the valuation of nature’s contribution to quality of life. Finally and crucially, the CF goes further than any previous initiative in the international environmental science–policy interface in its explicit, formal incorporation of knowledge systems other than western science, in an unprecedented effort towards crosscultural and crossdisciplinary communicability in the search for options and solutions.

**Box 1. The Main Elements of the IPBES Conceptual Framework [20,21]**

- **Nature** here refers to the natural world, with an emphasis on biodiversity and ecosystems. Nature has values related to the provision of benefit to people, and also intrinsic value, independent of human experience.
- **Anthropogenic assets** refers to knowledge, technology, financial assets, built infrastructure, etc.
- **Nature’s benefits to people** are all the benefits (and detriments or losses) that humanity obtains from **nature**. By definition, all **nature’s benefits** have human value, which can range from spiritual inspiration to market value. **Nature** provides some benefits to people directly without the intervention of society (e.g., oxygen). Most benefits, however, depend on the joint contribution of **nature** and **anthropogenic assets**, e.g., fish need to be caught to act as food.
- Drivers of change refers to all those external factors that affect **nature, anthropogenic assets, nature’s benefits to people**, and **good quality of life**. The CF includes drivers of change as two of its main elements: **institutions and governance systems and other indirect drivers** and **direct drivers** (both natural, such as earthquakes and tropical cyclones; and anthropogenic—e.g., habitat conversion, chemical pollution).
  - **Institutions and governance systems and other indirect drivers** are the root causes of the **direct anthropogenic drivers** that affect **nature**. They include systems of access to land, legislative arrangements, international regimes such as agreements for the protection of endangered species, and economic policies.
  - **Direct drivers**, both natural and anthropogenic, affect nature directly. The **direct anthropogenic drivers** are those that flow from **human institutions and governance systems and other indirect drivers**. They include positive and negative effects, e.g., habitat conversion (e.g., degradation or restoration of land and aquatic habitats), climate change, and species introductions. Direct natural drivers (e.g., volcanic eruptions) can directly affect **nature, anthropogenic assets**, and **quality of life**, but their impacts are not the main focus of IPBES.
- **Good quality of life** is the achievement of a fulfilled human life. It is a highly values-based and context-dependent element comprising multiple factors such as access to food, water, health, education, security, cultural identity, material prosperity, spiritual satisfaction, and freedom of choice. A society’s achievement of **good quality of life** and the vision of what this entails directly influences **institutions and governance systems and other indirect drivers** and, through them, all other elements. **Good quality of life**, also indirectly shapes, via institutions, the ways in which individuals and groups relate to **nature**.

Placed at the heart of an intergovernmental process, the CF is the result of political negotiation, but it goes beyond that. The consultative construction process that converged in the model adopted by the IPBES Second Plenary was rich in discussion and conflict on epistemological and methodological, as well as political, grounds. A major breakthrough during this process was to allow different knowledge systems to define the six elements according to their own categories. Previously, there had been a struggle to find a single word or phrase to capture the essence of each element in a way that respected the range of utilitarian, scientific, and spiritual values that makes up the diversity of human views of nature. The CF is now a kind of “Rosetta Stone” (see Box 2) for biodiversity concepts that highlights the commonalities between very diverse value sets and seeks to facilitate crossdisciplinary and crosscultural understanding. For example, the CF element **nature** includes scientific concepts such as species diversity, ecosystem structure and functioning, the biosphere, the evolutionary process and humankind’s shared evolutionary heritage (shown in green). For indigenous knowledge systems, **nature** includes different concepts such as “Mother Earth” and systems of life (for indigenous peoples of the South American Andes), and other holistic concepts of land and water, such as those held in the South Pacific islands, which include physical environment, non-human living organisms, living people, ancestors and their traditions (blue). Of course, a perfect alignment between concepts from different knowledge systems is probably unattainable. Instead, the framework provides common ground for basic working understanding and coordinated action towards tackling the biodiversity crisis. The broad crosscultural categories indicated as headlines of the boxes in Fig. 1 (in larger black font) should be transparent and important for all stakeholders involved and are proposed as standard for every assessment to be carried out by IPBES. Within them, different activities may identify more specific subcategories, associated with knowledge systems and disciplines relevant to the task at hand, without losing view of their placement within the general picture. For example, there is a large philosophical and instrumental gap between the ways in which gifts of nature and ecosystem goods and services are conceptualized, valued, and used according to different worldviews, but both categories are concerned with the good and bad things that societies obtain from the natural world, in the vast majority of cases through the mediation of institutions, be they ancestral rights to land, national economic policies, or international biodiversity treaties. In this way, general questions and problems can be formulated in a way that is intelligible across stakeholders, although they may strongly differ in the relative importance of different drivers in causing the problems and in the best responses to them.

**Box 2. The Rosetta Stone**

The Rosetta Stone is a inscribed rock tablet discovered in Egypt in 1799, which holds the key to understanding Egyptian hieroglyphs (http://www.britishmuseum.org/explore). The top band consists of Ancient Egyptian hieroglyphs, the middle band of Demotic script, and the bottom band of Ancient Greek writing. The inscriptions are three translations of the same decree, issued in Memphis in 196 BC, affirming the royal cult of Ptolemy V. In the early years of the 19th century, the Greek inscription was used as the key to deciphering the others.

The intellectual and practical challenges involved in the implementation of the IPBES model will be formidable. A conceptual scaffolding such as the CF may not be sufficient for fulfilling the IPBES vision of bringing on board stakeholders across disciplines, cultures, and knowledge systems in the search of solutions. We argue, however, that an inclusive CF is a necessary condition towards the success of such a vision.

## Moving into Practice: A Call for Embracing the Framework

In early 2014, IPBES started to implement its work programme: a set of coordinated assessments, policy tools, and capacity building actions [14]. The two initial assessments of IPBES focus on pollination and pollinators associated with food production on one hand, and scenarios analysis and modelling of biodiversity and ecosystem services on the other. Many others will follow in the years to come. If the vision behind the CF is embraced by the thousands of participants representing different disciplines, knowledge systems, and stakeholder groups who will perform IPBES-related work in the upcoming years, it will likely change the manner in which assessments have been done so far. Rather than focusing on one particular box or arrow, the framework is meant to inspire the community in looking at issues in an integrated manner, in an effort to consider the full cycle of events from causes to solutions. This is likely to push all engaged parties well beyond their comfort zones, but it will be worth the effort.

For example, rather than focusing mostly on direct drivers of pollination change (such as habitat or climate change, landscape alteration, overuse of pesticides, or spread of pathogens), as recent scientific reviews of regional or global declines in pollinators [15–17] have done, the CF is meant to invite experts to look at the underlying causes of these direct changes, such as institutional drivers. In the spirit of the CF, the pollination assessment will further examine the impacts of pollinator declines on subsistence agricultural systems, which provide much of the food in some regions of the world and yet are under-represented in recent case studies. Assisted by the IPBES Task Force on Indigenous and Local Knowledge, it will also consider the trends observed by practitioners and their interpretations of such trends and whether local and indigenous knowledge can offer solutions. It will have to take on board state-of-the-art metapopulation and metacommunity ecology, spatial modelling, microbiology and engineering, as well as social and economic analysis of the supply and demand chains, to identify which aspects are part of the problem and which are part of the possible solutions. It will have to propose options for integrated changes to trade in domestic bees, pesticide use, and incentives for the conservation of patches of vegetation with their wild pollinators embedded in agricultural landscapes, and adapt these options to different regions of the world. Lastly, it will need to answer the question: what would be the best mechanisms to involve government agencies, civil society, and the private sector to explain and curb the declining trends in pollinators involved in food production?

## Pushing the Frontiers of Biodiversity Science

The ability of the CF to provide insight and support transformative action will be tested as IPBES undertakes its work programme. IPBES is not alone in its quest for more integrative, cross-paradigm, co-produced knowledge. The CF is only one concrete step within a more general thrust that now includes many national research agencies, international funding bodies, and some of the largest scientific networks in the world. What are the implications of the CF for the ways in which we will do science in the coming years? Will the need for convergence of different disciplines and knowledge systems to solve concrete practice and policy problems compromise the sharpness of disciplinary science? The risk exists, but we argue that the opportunities greatly outweigh it. Disciplines will not necessarily need to become more superficial, or abandon their specific tools or their internal validity criteria [5]. Instead, they are likely to find novel questions worthy of all their analytical power. Indeed, one of the most crucial features of the CF is the way in which priority questions are generated and the way in which possible explanations and solutions are identified and put forward for practical implementation. As has happened many times through history, these new contexts of discovery and application, rather than blunting the cutting edge of science, will need it to be at its sharpest.

## Abbreviations

CF: Conceptual Framework
IPBES: Intergovernmental Platform on Biodiversity and Ecosystem Services
IPCC: Intergovernmental Panel on Climate Change
ILK: indigenous and local knowledge
